# Epidemic Characteristics, Spatiotemporal Pattern, and Risk Factors of Other Infectious Diarrhea in Fujian Province From 2005 to 2021: Retrospective Analysis

**DOI:** 10.2196/45870

**Published:** 2023-11-30

**Authors:** Yixiao Lu, Hansong Zhu, Zhijian Hu, Fei He, Guangmin Chen

**Affiliations:** 1 School of Public Health (Shenzhen) Shenzhen Campus of Sun Yat-sen University Shenzhen China; 2 Fujian Provincial Center for Disease Control and Prevention The Practice Base on the School of Public Health Fujian Medical University Fuzhou China; 3 Department of Epidemiology and Health Statistics School of Public Health Fujian Medical University Fuzhou China

**Keywords:** other infectious diarrhea, spatiotemporal pattern, disease cluster, epidemiological trends, spatial autocorrelation, meteorological factors, environmental factors

## Abstract

**Background:**

Other infectious diarrhea (OID) continues to pose a significant public health threat to all age groups in Fujian Province. There is a need for an in-depth analysis to understand the epidemiological pattern of OID and its associated risk factors in the region.

**Objective:**

In this study, we aimed to describe the overall epidemic characteristics and spatiotemporal pattern of OID in Fujian Province from 2005 to 2021 and explore the linkage between sociodemographic and environmental factors and the occurrence of OID within the study area.

**Methods:**

Notification data for OID in Fujian were extracted from the China Information System for Disease Control and Prevention. The spatiotemporal pattern of OID was analyzed using Moran index and Kulldorff scan statistics. The seasonality of and short-term impact of meteorological factors on OID were examined using an additive decomposition model and a generalized additive model. Geographical weighted regression and generalized linear mixed model were used to identify potential risk factors.

**Results:**

A total of 388,636 OID cases were recorded in Fujian Province from January 2005 to December 2021, with an average annual incidence of 60.3 (SD 16.7) per 100,000 population. Children aged <2 years accounted for 50.7% (196,905/388,636) of all cases. There was a steady increase in OID from 2005 to 2017 and a clear seasonal shift in OID cases from autumn to winter and spring between 2005 and 2020. Higher maximum temperature, atmospheric pressure, humidity, and precipitation were linked to a higher number of deseasonalized OID cases. The spatial and temporal aggregations were concentrated in Zhangzhou City and Xiamen City for 17 study years. Furthermore, the clustered areas exhibited a dynamic spreading trend, expanding from the southernmost Fujian to the southeast and then southward over time. Factors such as densely populated areas with a large <1-year-old population, less economically developed areas, and higher pollution levels contributed to OID cases in Fujian Province.

**Conclusions:**

This study revealed a distinct distribution of OID incidence across different population groups, seasons, and regions in Fujian Province. Zhangzhou City and Xiamen City were identified as the major hot spots for OID. Therefore, prevention and control efforts should prioritize these specific hot spots and highly susceptible groups.

## Introduction

### Background

Diarrheal disease is the second leading cause of malnutrition and death in children aged <5 years, with an estimated 1.7 billion yearly cases of childhood diarrheal disease worldwide. China ranked 11th among countries with the highest number of diarrheal deaths among children aged <5 years [1]. Infectious diarrhea is caused by a host of bacterial, viral, and parasitic organisms [2], and other infectious diarrhea (OID) was defined as infectious diarrhea other than cholera, dysentery, and typhoid or paratyphoid fever [3]. In China, bacterial and viral pathogens, such as *Shigella*, rotavirus, enterocathartic *Escherichia coli*, *Campylobacter jejuni*, and *Salmonella*_,_ are the most commonly identified pathogens associated with OID [4].

OID incidence varies by region in China because of the broad spectrum of pathogens; sociodemographic, environmental, and meteorological factors; hygiene; and living habits [5-7]. Moreover, the seasonal pattern and spatial distribution of OID also exhibit regional variations [8,9]. Understanding the role of environmental and meteorological exposures in these variations is critical for developing targeted public health interventions. Therefore, our study sought to investigate the association between county-level environmental and meteorological factors and the occurrence of OID in Fujian Province.

Analyzing a substantial, 17-year time frame allowed us to capture temporal variations, overall trends, and seasonal fluctuations in OID incidence, facilitating the identification of disease patterns over time and potential seasonality drivers, such as specific meteorological factors. For instance, temperature has been linked to increased diarrhea risk, particularly for diarrhea caused by bacterial pathogens [10,11], whereas heavy rainfall following dry periods may exacerbate incidence [12]. In addition, long-term data analysis provides a comprehensive understanding of epidemiological dynamics and aids in the identification of preventive strategies and interventions.

Spatial clustering methods, such as spatial scan statistics, local indicators of spatial association (LISA), and the Getis-Ord local Gi*(d) statistic, are commonly used for predicting disease risk and examining cluster characteristics based on location, size, and disease incidence [13]. In our study, we aimed to address gaps in OID epidemiology in Fujian Province by comprehensively examining its spatiotemporal distribution and identifying local clusters. We used a combined approach that integrated LISA and spatial scan statistics. This combination ensures a comprehensive identification of the different aspects of spatial patterns and logically consistent outcomes [14-16].

Moreover, recognizing the potential spatial clustering issues of OID, our study adopted a multilevel analysis to investigate the influence of sociodemographic and environmental factors. Specifically, we explored the emerging concern in China regarding potential human exposure to heavy metals through the consumption of contaminated food. Studies conducted in coastal areas have highlighted the primary exposure of soils and foods to highly toxic pollutants, such as arsenic, cadmium, and lead [17,18]. Soil and food pollution resulting from mining, industrialization, and agricultural activities can exacerbate this problem, leading to weakened immune systems [19] and increased susceptibility to diarrheal pathogens.

Southern China, including Fujian Province on the southeastern coast, experiences a typical monsoon climate characterized by high humidity, temperature, and rainfall. This is the region where the majority of OID-related public health emergencies have been documented [20]. In addition, factors such as rapid urbanization, the development of forestry and mining, and prevalent raw seafood eating habits have emerged as potential contributors to the recent rise in OID incidence. Despite the significant burden of OID and the consistent ranking of OID among the top 5 class C notifiable diseases in Fujian, a critical gap persists in the understanding of its epidemiological pattern in this region [21]. Although several studies have investigated OID distribution in the southeastern coastal areas of China [22,23], to the best of our knowledge, no comprehensive and systematic research has been conducted on the spatial distribution and clustering of OID at the county level in Fujian. Therefore, there is a need for an in-depth analysis to understand the epidemiological pattern of OID and its associated risk factors in the region.

### Objectives

The objective of this study was 2-fold. First, we aimed to provide an overall description of the epidemic distribution patterns and spatiotemporal distribution of OID in Fujian Province from 2005 to 2021. Second, we aimed to explore the linkage between sociodemographic and environmental factors and the occurrence of OID within the study area. By investigating these factors, we sought to enhance our understanding of the epidemiological pattern of OID and inform targeted interventions and preventive strategies to mitigate the impact of OID on public health outcomes in Fujian Province.

## Methods

### Study Setting

**Figure 1:**
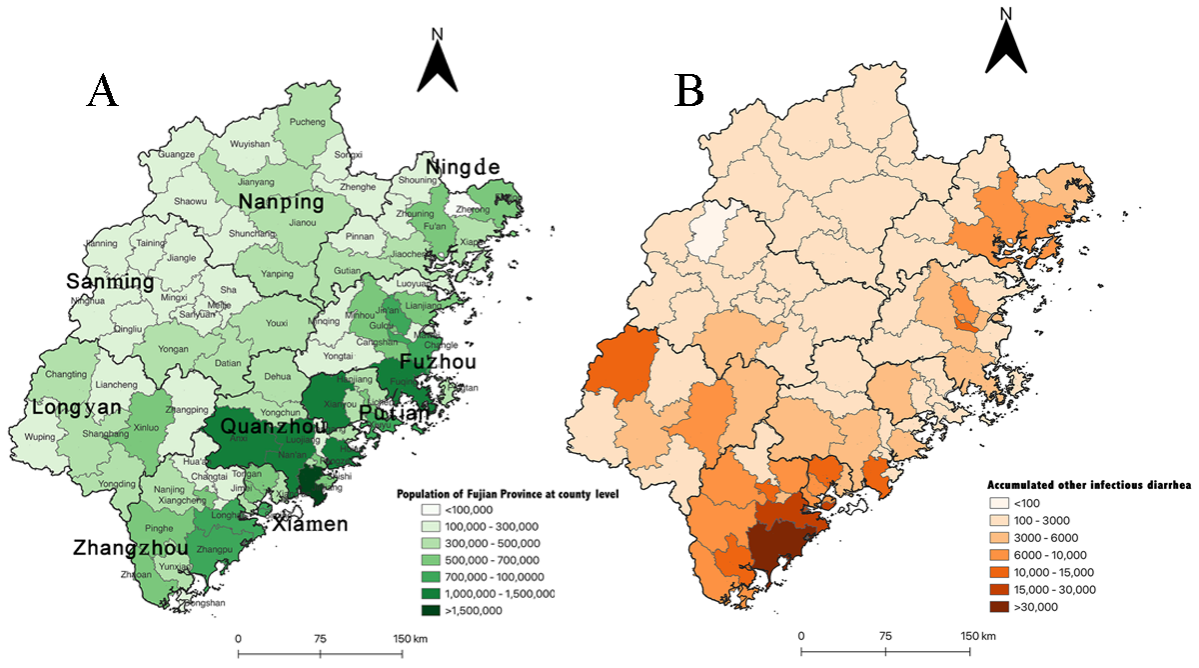
Reported other infectious diarrhea cases at the county level in Fujian Province from January 1, 2005, to December 31, 2010. (A) Population distribution. (B) Accumulated other infectious diarrhea distribution.

Fujian Province is located on the southeastern coast of China (23°33′–28°20′N, 115°50′–120°40′E) and consists of 6 coastal prefecture-level cities (Nindea, Fuzhou, Putian, Quanzhou, Xiamen, and Zhangzhou) and 6 mountainous cities (Nanping, Sanming, and Longyan), including 85 county-level divisions (Figure 1).

### Data Sources

The original data on OID in Fujian Province from 2005 to 2021 were obtained from the reporting subsystem of the China Information System for Disease Control and Prevention. OID cases were reported by medical institutions at all levels using the standard report card for infectious diseases, which includes information such as age, sex, occupation, date of onset, classification of disease, registration of residence, and residential address. Residential type is determined by the registration of residence and residential address. Permanent residents are individuals whose registration of residence and residential address are from the same county. The floating population inside city consists of individuals from different counties but the same city. The floating population inside province comprises individuals from different counties and cities within the same province, whereas the floating population outside province includes individuals whose registration of residence is outside Fujian Province. Cases of OID were diagnosed by physicians based on the history of exposure, clinical manifestation, and laboratory testing following the criteria issued by the National Health Commission of the People’s Republic of China (WS 271-2007) [24]. Clinically diagnosed cases refer to those with a clinical diagnosis alone, whereas confirmed cases refer to those with both a clinical diagnosis and a pathogen diagnosis.

A total of 402,727 cases were registered in the China Information System for Disease Control and Prevention between January 1, 2005, and December 31, 2021. Suspected cases without a confirmed diagnosis were excluded from the analysis data set, leaving 388,636 confirmed OID cases for inclusion in this study.

The population data used for calculating the incidence rate were sourced from the Fujian Statistical Yearbook, which is officially published on a yearly basis by the Fujian Provincial Bureau of Statistics. County-level data were additionally collected, including demographic factors (permanent residents, urban/rural population, <1-year-old population, and <5-year-old population), economic factors (gross domestic product [GDP] per capita; primary, secondary, and tertiary sector GDP; urbanization level; and highway density), and environmental factors (arsenic, cadmium, chromium, copper, lead, and zinc contents of the soil and fine atmospheric particles with a diameter of ≤2.5 μm [PM_2.5_]).

### Statistical Analysis

#### Time-Series Analysis

The characteristics of OID were summarized by frequency and proportion, and the chi-square test was conducted and odds ratio was calculated to evaluate the differences between subgroups, with a significance level of *P*<.05. A time-series analysis based on the additive decomposition model was performed to estimate the seasonal effects on the reported OID cases in Fujian Province from 2005 to 2021 using the following formula: Xt = seasonal + trend + random. Xt is the number of OID cases, and trend is time expressed in weeks.

To investigate the short-term effects of meteorological factors on OID, we collected daily data on average temperature (°C), maximum temperature (°C), minimum temperature (°C), atmospheric pressure at sea level (mm Hg), average relative humidity (%), and precipitation (mm) from February 2005 to December 2021. The meteorological data were obtained from the daily meteorological data set publicly released by the website Reliable Prognosis [25]. The website provides weather data collected from ground weather stations through the International Free Exchange System for Meteorological Data. The missing data points were imputed using the mean of neighboring values.

To capture the nonlinear relationships between meteorological factors and OID notification cases, we used the generalized additive model (GAM) [26]. We first aggregated weekly values of meteorological factors from the daily data and then averaged the weekly mean values in Fujian Province to derive weekly variables. Then, we detrended the underlying patterns and deseasonalized the weekly OID data. We used a nonparametric spline fitting response model based on a GAM to estimate the effect of meteorological factors on OID, using a Poisson model with the following formula: y = α + s(x1) + s(x2) +... + s(xm) + ε, incorporates detrended OID data (y); the model intercept (α); and smooth terms (s(x1), s(x2), and s(xm)) for each predictor variable; in addition, a cyclic cubic regression spline basis function (ε) is specified for the time variable. To select smoothing parameters in the GAM, we used the restricted (or residual) maximum likelihood method because the sample size was small.

#### Spatial Autocorrelation Analysis

The global Moran index was used to detect the spatial autocorrelation of OID cases in Fujian Province [27]. Significant positive spatial autocorrelation of OID cases implies that the distribution of OID cases is more spatially aggregated than a random underlying spatial process. The Moran index is calculated using the following equation: *I* = (n/*S*_0_​*)*​(∑^n^*_i_*_=1_​∑^n^*_j_*_=1_​*W_i_*_,_*_j_*​*Z_i_*​*Z_j_*​)/(∑^n^*_i_*_=1_​*Z_i_*^2^​), where *Z_i_* is the deviation of an attribute for feature *i* from its mean (*x_i_* –`*X*), *W_i,j_* is the spatial weight between features *i* and *j*, *n* is the total number of features, and *S_o_* is the aggregate of all spatial weights.

The global Moran I ranges from −1 to 1, with 0 indicating the null hypothesis of no clustering. A higher positive value of Moran I indicates the clustering of OID cases, whereas a lower negative value implies that neighboring areas are characterized by dissimilar OID cases [28]. The queen contiguity method was applied to define a weight matrix specifying the spatial relationships in consideration of the irregularity in the shapes and sizes of county-level divisions in Fujian [29].

In addition to global measures, local variations of OID cases’ spatial patterns were analyzed using a version of local Moran I, and LISA was computed for each location [30]. LISA was performed with 999 permutations to find core clusters and outliers of counties with extreme OID cases unexplained by random variation. As a result, LISA detects clusters as “hot spots” (high-high), “cold spots” (low-low), and “spatial outliers” (high-low or low-high) [31]. A *P* value <.05 for the cluster or outlier is considered statistically significant.

#### Retrospective Space-Time Scan Statistics

The SatScan software (version 9.6) applying the Kulldorff method of retrospective space-time analysis was used to identify potential clusters with high rates that significantly exceeded the OID incidence of nearby regions (*P*<.05). The space-time scan statistics use geographical information and time-periodic variable to explore temporal persistence and possible spatial accumulation. An infinite number of discrete, cylindrical windows are created by the space-time scan, with a circular geographic base and height corresponding to time [32]. Each cylindrical window was evaluated as a potential OID space-time cluster. Assuming that the data followed a Poisson distribution, the distribution and statistical significance of the space-time clusters were analyzed through Monte Carlo replication under the null hypothesis with the default 999 replications to ensure adequate power for defining clustering. The most likely cluster and a number of secondary potential clusters were reported. The relative risk of OID in each cluster was calculated to evaluate the risk of OID in the clustered areas using the following formula [33]: RR= (c/e)/((C-c)/(C-e)), where *c* is the total number of cases in a cluster, *e* is the number of expected cases in a cluster, and *C* is the total number of observed cases in Fujian Province.

**Figure 2:**
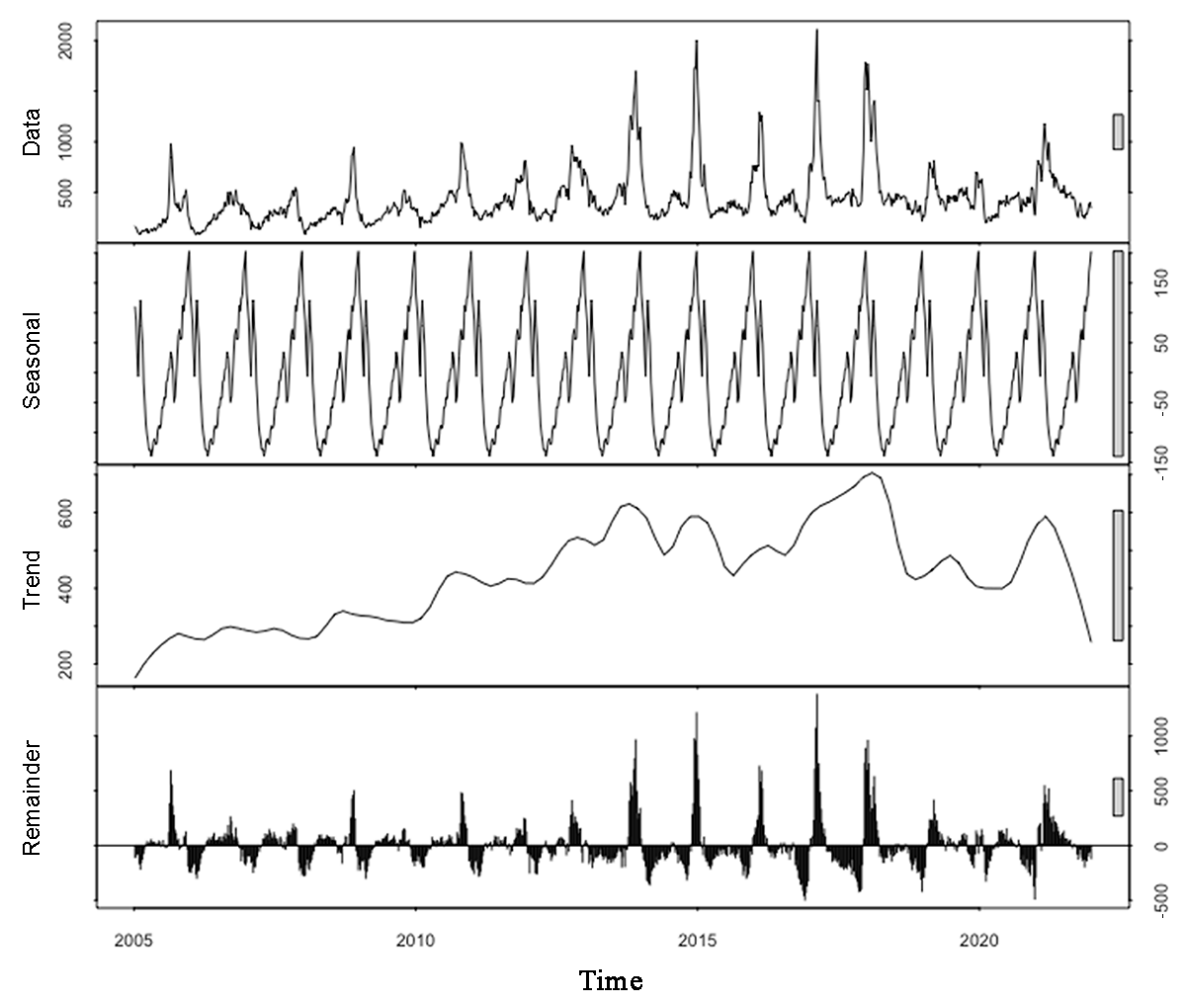
The trend and seasonal decomposition analysis of other infectious diarrhea in Fujian Province (from January 1, 2005, to December 31, 2021).

The OID data from 2005 were used to determine the spatial scanning windows by increasing from 1% to 50% of the population at risk, and the value was limited to 17%, considering no overlap of candidate clusters and covering the largest high-incidence geographic areas [34]. Given that the periodicity of the reported OID cases was approximately 2 to 3 months (Figure 2), the temporal scanning window was set to 90 days. Moreover, each candidate cluster had to include no less than 2 cases and have a minimum of 2 days. Age and sex were used as 2 covariate variables for covariate adjustment in the scan statistics [35]. The retrospective space-time scan statistics were applied for each year of OID in Fujian Province from 2005 to 2021.

#### Spatial Regression Analysis

Ordinary least regression (OLS) was used to determine the relationship between OID incidence (of each county) and each of the potential influencing factors (including demographic, economic, and environmental factors). Considering the spatial variation of the regression coefficient, geographical coordinates were included as parts of the regression parameters. Geographical weighted regression (GWR) is an effective method for determining spatial variables by considering local-scale features of geographical elements (county level in this study). The GWR is computed as follows [36]:

*y_i_* ​= ∑*_k_*​*β_k_*​(*u_i_*​, *v_i_*​)*x_k_*_,_*_i_* ​+ *ε_i_*​.

Corrected Akaike information criterion (AICc) [37] and *R*^2^ values, which consider model complexity and goodness of fit, were used to evaluate the OLS and GWR models [38].

Generalized linear mixed model (GLMM) [39] was used to investigate the factors influencing the occurrence of clustered types of OID cases, distinguishing whether the OID cases fall within the clustered areas (coded as “1”) or outside of them (coded as “0”) at both individual and county levels. The individual-level variables considered in the analysis were sex (male or female), age (<1 year, 1-5 years, or ≥5 years), and residential type. For the county-level variables (as mentioned earlier), we selected predictors based on the variance inflation factor to mitigate multicollinearity among the model terms. To evaluate the goodness of fit, we used Akaike information criterion (AIC) and Schwarz Bayesian information criterion [40]. Among the different models tested, the model with the lowest AIC and Bayesian information criterion values was deemed to be the most suitable and informative.

All statistical analyses were performed using RStudio (version 1.4.1717) [41]; time-series regression analysis was performed using the *mgcv* R package, spatial autocorrelation analysis was performed using the *spdep* R package, geographically weighted regression was performed using the *GWmodel* R package, GLMM was performed using the *lme4* R package, and the choropleth maps representing the detected disease clusters were generated using QGIS 3.20.2 Odense.

### Ethical Considerations

The study was approved by the ethical review board of the Fujian Center for Disease Control and Prevention (Fuzhou, China; number 2020-029), and it was performed in accordance with the principles of the Declaration of Helsinki. Consent to participate was not applicable because this study used OID surveillance data. All data were anonymized and kept confidential to protect the privacy of participants.

## Results

### Assessment of Case-Reporting Quality

The data set under analysis comprised 388,066 (99.9%) out of 388,636 cases with complete information on the following epidemiological variables: sex, date of birth, occupation, residential type, notification type, symptom onset date, date of diagnosis, and date of data entry. A total of 570 cases without detailed residential addresses were excluded for spatial and spatiotemporal analysis purposes. The reporting delay (between the date of diagnosis and date of data entry) was calculated for all 388,636 cases, and the median (IQR) delay was 0 (−375 to 1) days. We further identified 1879 cases with a negative reporting delay. However, for our subsequent analysis, we used the diagnosis date, rendering the impact of the quality of reporting delay insignificant.

### Characteristics of the Reported OID Cases

From January 1, 2005, to December 31, 2021, a total of 388,636 OID cases were reported from 85 county-level divisions (12 cities, 29 districts, and 44 counties) in Fujian Province; the reported OID cases were mainly from coastal areas (Figure 1). Table 1 summarizes the characteristics of the reported OID cases. Of the 388,636 patients with OID, 228,777 (58.9%) were male. The median (IQR) age of the patients was 1 (0-24) year, and 70.5% (273,988/388,636) of the patients were children aged <5 years; patients aged <1 year (108,818/388,636, 28%) accounted for the highest proportion of patients, followed by patients aged 1 to 2 years (88,220/388,636, 22.7%).

Table 2 presents a comparison of the characteristics of patients with OID aged <5 years and patients with OID aged >5 years. The differences in sex, notification type, and residential type between the 2 age groups were statistically significant (*P*<.001). Males aged <5 years had 1.56 times higher odds of being diagnosed with OID than those aged >5 years. Moreover, patients aged <5 years had 6.03 times higher odds of confirming OID through a clinical and pathogen diagnosis than those aged >5 years. In addition, within the floating population (both within city and province), children aged <5 years had 3.18 and 1.44 times higher odds, respectively, of having OID than those aged >5 years.

**Table 1:** Characteristics of other infectious diarrhea in Fujian Province from 2005 to 2021.

Characteristics				Patients with OID (N=388,636), n (%)
**Sex**				
	Male		228,779 (58.9)	
	Female		159,857 (41.1)	
**Age (years)^a^**				
	0		108,675 (28)	
	1		88,230 (22.7)	
	2		30,328 (7.8)	
	3		15,736 (4)	
	4		8713 (2.2)	
	5		22,440 (5.8)	
	15		49,080 (12.6)	
	40		37,403 (9.6)	
	≥60		28,031 (7.2)	
**Notification type**				
	Confirmed case		140,140 (36.1)	
	Clinically diagnosed case		248,496 (63.9)	
**Occupation**				
	Diaspora children		245,863 (63.3)	
	Farmer		59,786 (15.4)	
	Student		19,520 (5)	
	Unemployed or housework or retired		18,893 (4.9)	
	Childcare		14,648 (3.8)	
	Migrant worker		1691 (0.4)	
	Medical staff		709 (0.2)	
	Others		20,755 (5.3)	
	Unknown		6771 (1.7)	
**Residential type**				
	Permanent residents		275,768 (71)	
	**Floating population**		112,868 (29)	
		Inside city	94,159 (24.2)	
		Inside province	11,790 (3)	
		Outside province	6919 (1.8)	

^a^Age was grouped under 1 year, 2 years, 3 years, etc.

**Table 2:** Comparison of patients aged <5 years and patients aged >5 years in Fujian Province from 2005 to 2021^a^.

Characteristics				Patients aged <5 years (n=251,682), n (%)		Patients aged >5 years (n=136,954), n (%)		OR^b^ (95% CI)		*P* value	
**Sex**											<.001
	Male			158,439 (63)		70,340 (51.4)		1.56 (1.53-1.59)			
	Female			93,243 (37)		66,614 (48.6)		0.64 (0.63-0.66)			
**Notification type**											<.001
	Confirmed case			122,567 (48.7)		17,573 (12.8)		6.03 (5.92-6.15)			
	Clinically diagnosed case			129,115 (51.3)		119,381 (87.2)		0.11 (0.11-0.11)			
**Residential type**											<.001
	Permanent residents			161,809 (64.3)		113,959 (83.2)		0.3 (0.29-0.31)			
	**Floating population**										
		Inside city	77,612 (30.8)		16,547 (12.1)		3.18 (3.13-3.23)				
		Inside province	8436 (3.4)		3354 (2.4)		1.44 (1.36-1.53)				
		Outside province	3825 (1.5)		3094 (2.3)		0.65 (0.61-0.69)				

^a^Statistical significance (chi-square test) between patients aged <5 years and patients aged >5 years was set at *P*<.05.

^b^OR: odds ratio.

### Incidence and Trend Analysis of the Reported OID Cases

The average annual incidence of OID was 60.3 (SD 16.7) per 100,000 people; the annual incidence of OID peaked in 2017 (at 88.3) and was lowest in 2005 (at 39.5). Table 3 shows the incidence of OID by sex and age. The highest OID incidence was reported in individuals aged <1 year (1362.1 per 100,000 people), followed by individuals aged <2 years (1055.5 per 100,000 people). Low incidence was observed in the individuals aged 15 to 40 years and individuals aged 40 to 60 years (19.2 and 21.2 per 100,000 people, respectively). The average annual incidence among males (71.4 per 100,000) was significantly higher than that among females (52.5 per 100,000; *P*<.05). Specifically, in the age groups >5 and ≥60 years, the incidence among males was higher than that among females, whereas in the age groups 15 and 40 years, the incidence among females was significantly higher than that among males.

**Table 3:** The incidence of other infectious diarrhea in Fujian Province, stratified by age and sex, from January 1, 2005, to December 31, 2021.

Age (years)^a^	Total		Male		Female	
	Reported cases, n (%)	Average annual incidence (per 100,000)	Reported cases, n (%)	Average annual incidence (per 100,000)	Reported cases, n (%)	Average annual incidence (per 100,000)
0	108,673 (28)	1265.6	70,553 (30.8)	1589.4	38,122 (23.8)	992.4
1	88,229 (22.7)	1042.5	54,575 (23.9)	1202.5	33,655 (21.1)	857.4
2	30,328 (7.8)	354.1	18,514 (8.1)	406.9	11,814 (7.4)	304.2
3	15,736 (4)	197.9	9489 (4.1)	224.1	6247 (3.9)	171.8
4	8713 (2.2)	114.0	5308 (2.3)	131.6	3405 (2.1)	98.6
5	22,440 (5.8)	28.8	14,233 (6.2)	36.2	8207 (5.1)	24.3
15	49,080 (12.6)	18.5	24,276 (10.6)	17.8	24,804 (15.5)	19.1
40	37,403 (9.6)	20.3	17,912 (7.8)	19.4	19,491 (12.2)	21.7
≥60	28,031 (7.2)	35.1	13,919 (6.1)	37.3	14,112 (8.8)	35.5
All	388,633 (100)	60.3	228,777 (100)	70.0	159,856 (100)	51.3

^a^Age was grouped under 1 year, 2 years, 3 years, etc.

The weekly accumulated number of reported OID cases in Fujian Province presented a gyrating upward trend from 2005 to 2018, reaching a peak around July to August in 2017; showed a downward trend until the beginning of 2020; and then rose again (Figure 2, “trend”). The seasonal decomposition analysis revealed an annual seasonal pattern (Figure 2, “seasonal”). However, the peak months varied throughout the study period. In 2005, 2006, 2010, 2012, and 2014, most OID cases were reported in autumn (September-November); conversely, in 2007, 2008, 2009, 2013, 2015, 2016, 2017, 2018, and 2020, the peak period was in winter (December-February). In 2019 and 2021, the peak month was March (Figure 2, “data”). There was a clear seasonal shift in the peak month for OID cases from autumn to winter or spring between 2005 and 2020.

Using meteorological data collected from February 2005 to December 2021 (encompassing 882 weeks), the association between the number of OID cases and meteorological factors was examined. The GAM revealed that atmospheric pressure at sea level (*P*<.001), humidity (*P*<.001), precipitation (*P*=.01), and maximum temperature (*P*=.02) were significantly linked to detrended OID data, whereas average temperature, minimum temperature, and time (weeks) were not significantly related. The results indicated that a higher maximum temperature, atmospheric pressure, humidity, and precipitation were associated with higher detrended OID data. The model explained 12.6% of the deviance in the data, and the adjusted *R*^2^ value was 0.112. A summary of the GAM results, along with its diagnostics, is presented in Table 4.

**Table 4:** GAM^a^ of meteorological factors and other infectious diarrhea in Fujian Province (from February 4, 2005, to December 31, 2021).^b^

Variable (smoothed)	*df*	Reference *df*	*P* value
Time	0.005	8	.59
Average temperature	1.024	1.038	.22
Maximum temperature	2.458	3.109	.02^c^
Minimum temperature	1	1.001	.99
Atmospheric pressure	6.052	7.294	<.001^d^
Relative humidity	2.62	3.349	<.001^d^
Precipitation	1.005	1.01	.01^c^
Adjusted *R*^2^	0.112	N/A^e^	N/A
Deviance explained, %	12.60	N/A	N/A
REML^f^	5216.9	N/A	N/A
Number of weeks	882	N/A	N/A

^a^GAM: generalized additive model.

^b^Intercept: estimate=0.095, SE 3.065; *P*=.98.

^c^*P*<.05.

^d^*P*<.001.

^e^N/A: not applicable.

^f^REML: restricted maximum likelihood.

### Spatial and Spatiotemporal Analysis of the Reported OID Cases

**Figure 3:**
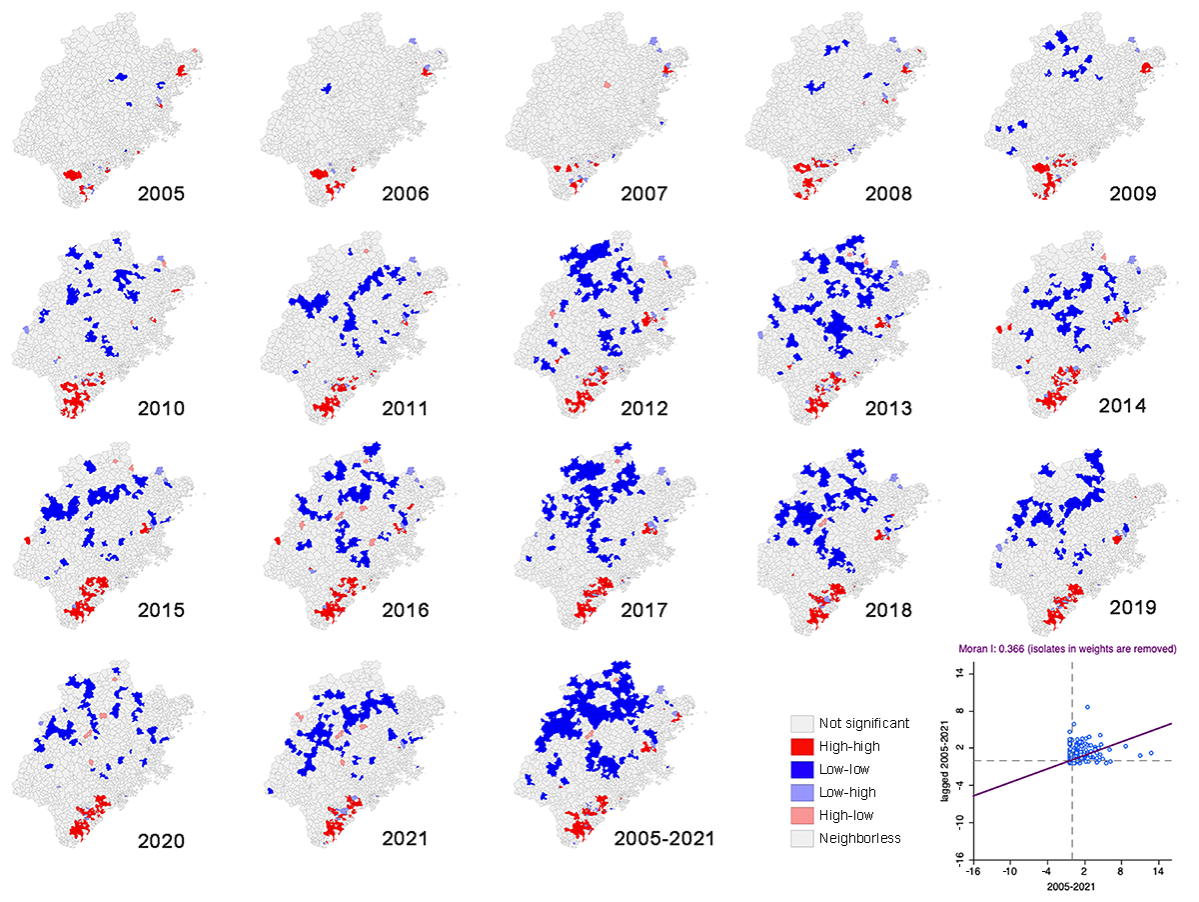
Moran scatterplot and local indices of spatial association cluster map of other infectious diarrhea at county level in Fujian Province (from January 1, 2005, to December 31, 2021). A higher resolution version of the figure is available in Multimedia Appendix 1.

The spatial autocorrelation analysis using the global Moran I demonstrated a positive correlation (0.20-0.32, *P*<.001) of OID cases from 2005 to 2020 (Table 5), indicating a significant overall spatial autocorrelation. The localities with clusters of OID cases were identified using local Moran I (LISA). Figure 3 displays the Moran scatterplot for the 17 study years (2005-2021), which reveals that most event points were gathered in the upper-right (high-high) quadrant, confirming the existence of positive spatial autocorrelation among the counties based on OID cases. The LISA cluster map depicted the locations of the high clusters (high surrounded by high), low clusters (low surrounded by low), and spatial outliers (mixture of high and low clusters) in the OID cases.

In Figure 3, the LISA map for all study years (2005-2021) shows that some counties on the south coast of Fujian Province exhibited high-high clusters, whereas the northwest and middle parts were found to be covered by low-low clusters. The yearly LISA map revealed outstanding spatial clusters of OID cases covering specific areas each year. From 2005 to 2007, the clustered areas with the highest density of reported OID cases (high-high) were primarily concentrated in Zhangzhou City, and in 2008, these areas drifted northeastward to Xiamen City and Quanzhou City. Other clustered areas with high OID rates appeared in Ningde City from 2005 to 2011 and in Fuzhou City from 2012 to 2019. Most of the high-low outliers were dispersed in the middle part of Fujian Province (Sanming City and Nanping City), mainly in 2016, 2020, and 2021; these high-low areas were generally surrounded by low-low clusters, which were mountainous areas. The low-high outliers appeared in the same location in Ningde City (northern part) from 2006 to 2018. The clusters and outliers were statistically significant based on *z* test (*P*<.05).

**Table 5:** The global Moran index of other infectious diarrhea in Fujian Province (from January 1, 2005, to December 31, 2021).

Year	Moran I	*z* score	*P* value
2005	0.22	12.20	<.001
2006	0.21	11.66	<.001
2007	0.24	12.88	<.001
2008	0.23	11.90	<.001
2009	0.30	15.51	<.001
2010	0.25	12.82	<.001
2011	0.23	12.00	<.001
2012	0.21	10.84	<.001
2013	0.22	11.24	<.001
2014	0.23	12.32	<.001
2015	0.28	14.49	<.001
2016	0.28	14.58	<.001
2017	0.32	16.55	<.001
2018	0.29	15.40	<.001
2019	0.26	13.87	<.001
2020	0.20	11.31	<.001
2021	0.31	16.53	<.001

Table 6 shows the most likely cluster of OID cases detected by retrospective space-time scan statistics from 2005 to 2020 in Fujian Province. The time frame was from September to November (2005, 2006, and 2010), mid-June to mid-September (2007), October to December (2008, 2009, and 2012-2014), June to August (2011), January to March (2015-2017), August to October (2018), July to September (2019), and mid-January to mid-April (2020). The average radius of the most likely clusters over the 17-year study period was 56.35 km. The average number of counties included in the most likely cluster was 9.76, with an average relative risk of 8.79.

**Figure 4:**
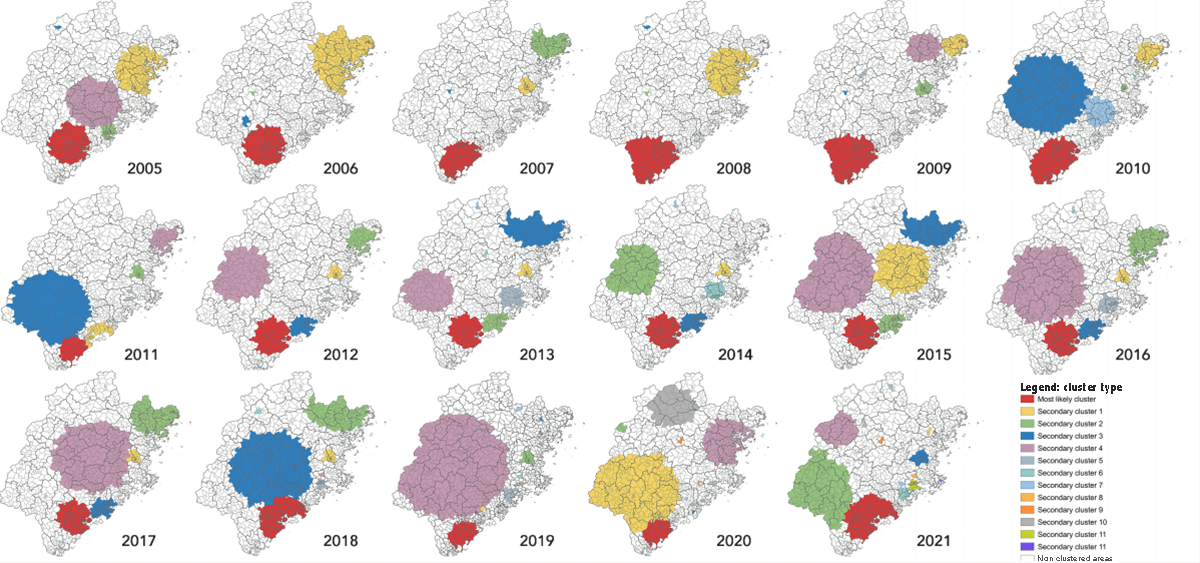
Annual spatial distribution of space-time clusters of other infectious diarrhea at county level in Fujian Province (from January 1, 2005, to December 31, 2021). A higher resolution version is available in Multimedia Appendix 2.

The most likely clusters were assembled in the southern part of Fujian Province, mainly in Zhangzhou City, Xiamen City, and Quanzhou City (Figure 4). The most likely cluster was detected in the north of Zhangzhou City and south of Xiamen in 2005 and 2006, respectively, and then moved southward to cover the entire region of Zhangzhou City between 2007 and 2010. Again, it moved northward to Xiamen City, covering the northern part of Zhangzhou City and the southern part of Quanzhou City.

**Figure 5:**
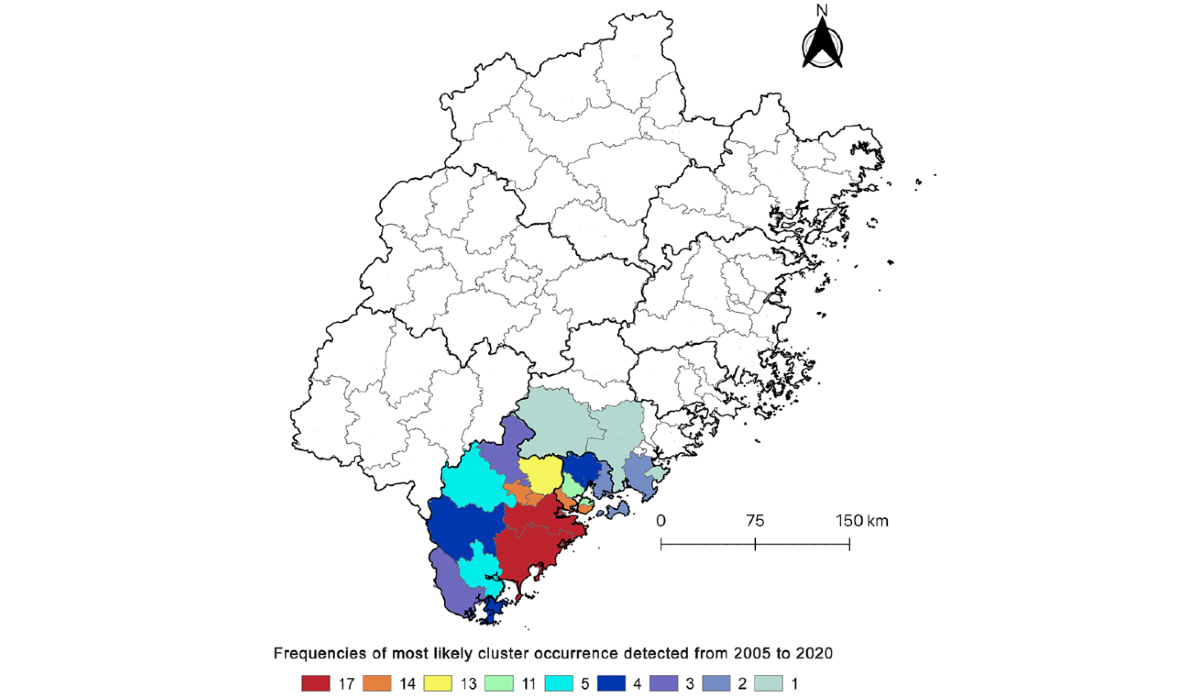
Frequency of most likely cluster occurrence at county level in Fujian Province (from January 1, 2005, to December 31, 2021).

Figure 5 illustrates the frequency of OID most likely clusters within districts based on retrospective space-time scan statistics. Two counties in Zhangzhou City (Zhangpu and Longhai) were detected 17 times (each year) using this method, and the median (IQR) frequency for the occurrence of most likely clusters per county was 4.5 (2.25-13.75) times. The southern regions of the province exhibited the highest frequency of OID clusters, whereas no most likely clusters were detected in the central, western, or northeast districts during the study period.

**Table 6:** The most likely cluster of other infectious diarrhea detected using space-time scan statistics in Fujian Province (from January 1, 2005, to December 31, 2021).

Year	Period	Radius (km)	Observed cases, n	Expected cases	Relative risk	*P* value	Total counties, n	Population, n
2005	September 5 to December 2	53.88	2716	531.0	6.12	<.001	13	5,550,594
2006	August 30 to November 27	53.88	2808	554.1	6.09	<.001	13	5,624,309
2007	June 17 to September 14	58.51	2218	492.2	5.11	<.001	9	4,409,866
2008	October 6 to December 31	97.44	3010	648.4	5.47	<.001	12	5,622,875
2009	October 3 to December 31	97.44	2319	648.4	4.02	<.001	12	5,680,749
2010	August 26 to November 23	72.07	3628	550.6	7.70	<.001	14	5,739,619
2011	May 29 to August 26	39.75	1327	91.0	15.47	<.001	3	1,202,354
2012	October 4 to December 31	45.67	2532	372.1	7.45	<.001	9	3,497,184
2013	October 6 to December 31	45.67	3158	455.5	7.58	<.001	9	3,521,250
2014	October 3 to December 31	45.67	2678	379.6	7.77	<.001	9	3,521,250
2015	January 1 to March 25	45.67	3356	322.8	11.87	<.001	9	3,443,638
2016	January 2 to March 31	45.67	3394	350.2	11.04	<.001	9	3,474,991
2017	January 2 to April 1	45.67	3859	468.4	9.16	<.001	9	3,505,793
2018	January 1 to March 29	63.89	6226	1228.5	6.07	<.001	14	9,479,996
2019	July 29 to October 26	39.75	1893	128.6	15.82	<.001	3	1,341,011
2020	June 28 to September 25	39.75	1630	104.4	16.85	<.001	3	1,341,996
2021	January 16 to April 15	67.54	5851	1186.8	5.92	<.001	16	11,084,758

### Risk Factors Related to OID Incidence

The relationship between OID incidence and the collected county-level sociodemographic factors was analyzed using a multivariate OLS regression model. The permanent resident population showed a significantly positive association. By contrast, the urban population, rural population, and <5-year-old population showed a significantly negative association with OID cases, and <1-year-old population was positively associated with OID cases, although not statistically significantly. Regarding economic factors, GDP per capita and primary sector’s GDP were positively associated with OID cases. By contrast, the secondary and tertiary sectors’ GDP and urbanization levels were negatively associated with OID cases. For environmental factors, the contents of arsenic, cadmium, chromium, copper, lead, and zinc in the soil were included in the OLS model, of which arsenic, chromium, and zinc were negatively associated, and the others were positively associated. Table 7 presents a summary of the multivariate OLS with model diagnostics.

A locally linear, nonparametric estimation using the GWR was used to cope with the OLS’s nonstationary nature. The adjusted *R*^2^ values rose from 0.389 to 0.405 (Table 7), even if the GWR did not improve the model fit from the OLS model according to the AICc values.

Table 8 and Table 9 summarized the results of GLMM. The response variable “clustered type” in the GLMM indicated whether the OID case is within the clustered areas (“1”) or outside of them (“0”). The clustered areas corresponded to counties that were detected at least once within the most likely clusters from the previous analysis. The GLMM adopts a binomial family with a logit link function, which is appropriate for analyzing binary outcomes. The maximum likelihood method (Laplace approximate) was used for estimation. The model demonstrated good fit, as indicated by AIC=24,672.6 and Bayesian information criterion=24,781.3. The results showed that population density, urbanization level, and copper content had positive estimates, suggesting that an increase in these variables was associated with a higher likelihood of OID cases being within the clustered areas. Conversely, arsenic, cadmium, and chromium contents had negative estimates, implying that a decrease in these variables was associated with a higher likelihood of OID cases being outside the clustered areas.

**Table 7:** Multivariate ordinary squares regression and geographic weighted regression model results of other infectious diarrhea in Fujian Province (from January 1, 2005, to December 31, 2021).^a^

Variable		Ordinary least regression (OLS) model		
		Β	SE	*P* value
**Population**				
	Permanent resident	0.050	0.022	.03^b^
	Urban	−0.046	0.021	.03^b^
	Rural	−0.049	0.022	.03^b^
	<1 year old	0.077	0.044	.08
	<5 year old	−0.024	0.011	.03^b^
**Economic factor**				
	GDP^c^ per capita	0.009	0.003	.004^d^
	Primary sector GDP	0.231	2.653	.93
	Secondary sector GDP	−3.582	0.995	<.001^d^
	Tertiary sector GDP	−2.292	0.881	.01^b^
	Urbanization (%)	−9.438	5.492	.090
**Environmental factor**				
	Arsenic content	−55.301	16.157	<.001^d^
	Cadmium content	0.363	1.984	.86
	Chromium content	−1.209	0.557	.03^b^
	Copper content	0.442	0.759	.56
	Lead content	0.019	0.025	.47
	Zinc content	−0.051	0.035	.15
	AICc^e^	1208.861	N/A^f^	N/A
	Adjusted *R*^2^	0.389	N/A	N/A
	AICc (GWR^g^)	1208.100	N/A	N/A
	Adjusted *R*^2^ (GWR)	0.405	N/A	N/A

^a^Intercept: estimate=495.003, SE 317.773; *P*=.12.

^b^*P*<.05.

^c^GDP: gross domestic product.

^d^*P*<.01.

^e^AICc: corrected Akaike information criterion.

^f^N/A: not applicable.

^g^GWR: geographical weighted regression.

**Table 8:** Fixed effects of generalized linear mixed model results of other infectious diarrhea in Fujian Province (from January 1, 2005, to December 31, 2021).

Coefficients	Estimate	SE	*z* value	*P* value
Intercept	−11.205	0.716	−15.64	<.001
Population density	2.342	0.101	23.21	<.001
Urbanization (%)	14.706	0.187	78.54	<.001
Arsenic content	−12.556	0.387	−32.47	<.001
Cadmium content	1.044	0.032	32.52	<.001
Chrome content	−7.059	0.122	−58.07	<.001
Copper content	1.944	0.169	11.49	<.001

**Table 9:** Random effects of generalized linear mixed model results of other infectious diarrhea in Fujian Province (from January 1, 2005, to December 31, 2021).

Random effects	Variances	SD
Residential types	0.163	0.404
Age group	0.002	0.049
Sex	0.003	0.056

## Discussion

### Principal Findings

In this study, we investigated the overall epidemic characteristics, spatiotemporal pattern, and risk factors associated with the occurrence of OID in Fujian Province. By analyzing 17 years of surveillance data, we identified the clustering of OID cases in Zhangzhou City and Xiamen City, with the clustering areas remaining relatively stable over time. Our findings also revealed that densely populated areas with a large <1-year-old population, less economically developed areas, and higher pollution levels were associated with higher OID incidence in the province. Moreover, high population density, urbanization level, and cadmium and copper contents in the soil could contribute to the clustering of OID.

The results highlighted the susceptibility of children aged <5 years; particularly, those aged 0 to 2 years accounted for a significant proportion (196,905/388,636, 50.7%) of the OID cases in Fujian Province. This finding aligns with previous research indicating their susceptibility to OID [42-44]. Viral pathogens such as rotavirus, adenovirus, norovirus, and Salmonella were the 4 most common pathogens in China [45]. Specifically, rotavirus infection increased rapidly in children aged <5 years [7,46]. Previous studies in Fujian Province have also emphasized the significance of rotavirus as a causative pathogen for acute gastroenteritis among children who are hospitalized [47].

Temporal analysis revealed a steady increase in OID cases from 2005 to 2017, with a clear seasonal shift in OID cases from autumn to winter/spring between 2005 and 2020. However, since 2020, there has been subsequent stagnation in case numbers, potentially linked to the impact of the COVID-19 pandemic. Behavioral interventions and policies implemented during the pandemic to suppress the transmission of respiratory infections [48] may have inadvertently reduced the transmission of other infectious diseases, including OID.

Our study found that maximum temperature, atmospheric pressure at sea level, average relative humidity, and precipitation are positively associated with the reported OID cases in Fujian, consistent with previous research in the coastal area of China [22,49,50]. Infectious diarrhea is often caused by microorganisms that are sensitive to environmental conditions [51], and higher temperatures, atmospheric pressure, and humidity may favor the survival and proliferation of microorganisms causing diarrhea [52]. Moreover, increased atmospheric pressure may reduce the dilution of airborne pathogens, leading to an increased concentration of pathogens and further increasing the likelihood of diarrhea transmission [44]. In addition, variations in rainfall and temperature can increase the risk of fecal contamination and diarrhea, particularly during heavy rainfalls and floods. The observed seasonal shift in OID cases from autumn to winter suggests a possible increase in viral infections as the main cause of OID during the winter, possibly owing to a more favorable environment for viral pathogen transmission [53]. However, this hypothesis needs further investigation.

Spatial analysis identified the presence of persistent “hot spots” of OID cases, mainly concentrated in Zhangzhou City and Xiamen City within the study period. These regions have a subtropical monsoon climate, with factors such as high humidity, temperature variation, heavy rainfall, and wind speed potentially facilitating OID transmission [8,22,42]. The predominant pathogen in these areas is rotavirus, which exhibits apparent seasonality during the autumn-winter season [47]. Other risk factors such as the consumption of raw seafood, which often contains pathogens such as rotavirus, norovirus, and *Vibrio parahaemolyticus*, have also been identified [54]. A study conducted in Taiwan on *Campylobacter* gastroenteritis revealed an increased risk of campylobacteriosis in children associated with seafood consumption [55].

In our study, almost 90% (15/17) of the most likely clusters (except for those in 2007 and 2011) were detected in the autumn and winter seasons, consistent with reported gastroenteritis outbreaks caused by norovirus [56]. These results suggest that viral pathogens and foodborne clustered infections are potential contributors to the identified OID clusters in the spatiotemporal analysis. Strengthening outbreak management capacities and implementing robust prevention and control measures in high-incidence areas are necessary. In addition, the high prevalence of human intestinal protozoa [57] and significant environmental pollution in both the land and adjacent sea areas [58] may be associated with the 2 most clustered counties (Zahngpu and Longhai) in Fujian Province found in this study. Furthermore, the clustered areas exhibited a dynamic spreading trend, expanding from the southernmost region to southeast and gradually southward over time. Therefore, close monitoring of the epidemic situation in the surrounding areas is essential.

Regression analysis indicated a positive association between OID cases and factors such as densely populated areas with a large >1-year-old population, less economically developed areas, and higher pollution levels. Children aged <5 years are susceptible to viral infectious diarrhea and are likely to further damage their health [59]. Countermeasures, including rotavirus vaccine [60], improvement of zinc nutriture [61], and dietary counseling [62], have been proven to reduce the high burden of childhood diarrhea. In coastal provinces, such as Fujian, living habits that involve frequent contact with seafood could be related to OID. Hygiene and cooked food education should be enhanced in high-risk areas, such as Zhangzhou City. Poor public health, poor water safety, fecal contamination, and environmental pollution pose obstacles to diarrhea prevention in economically underdeveloped areas [42]. Nevertheless, the impact of social factors on diarrhea remains uncertain [63]. Therefore, further research is needed to explore the major mechanisms underlying the association between infectious diarrhea and certain social factors.

The findings from our study underscore the need for implementing targeted prevention and control measures for OID in Fujian, with a particular focus on susceptible populations, such as young children. Furthermore, the health department should prioritize improving the living environments in Zhangzhou and Xiamen. Regular monitoring of the epidemic characteristics of infectious diarrheal diseases, incorporating meteorological factors into surveillance systems, and developing early warning models can enhance preparedness and response to outbreaks. This study’s findings contribute to the understanding of the epidemic characteristics of OID in Fujian Province and provide a vital foundation for informing future risk assessments and facilitating early warning predictions of this disease. Examining the impact of OID in Fujian Province can provide valuable epidemiological insights and effective prevention and control strategies that can be transferred to other regions and countries facing similar challenges.

### Limitations

Our study has several limitations. First, the lack of detailed information on the etiology of OID cases hinders further investigation into the pathogenesis of OID in infants and children as well as the identification of specific pathogen-related epidemic patterns. Hence, a province-wide pathogen monitoring and surveillance system is needed, which would provide valuable information for in-depth pathogen characterization. Second, the power of cylindrical scan statistics could be limited to the complex geographic landscape of Fujian Province. More flexible shapes of scan statistics or the use of higher-resolution geographical information can enhance the accuracy and precision of the analysis. Third, although the GAM used in our study captured some of the short-term effects of meteorological factors on OID cases, it explained only a small proportion of the overall variation. Therefore, more suitable forecasting models should be considered to enhance the predictive ability. Fourth, the regression models used to assess the association between OID and risk factors had inherent limitations. Furthermore, the lack of county-level meteorological and socioeconomic data, such as public health resources, further weakened the explanatory power of the regression model. Future research should incorporate predictive models that forecast the incidence of OID in the near and long term, considering the impact of preventive measures. This approach would highlight the importance of ongoing targeted interventions to control OID and improve the health and well-being of susceptible populations. Finally, owing to data limitations, we could not directly assess the influence of the COVID-19 pandemic and its subsequent nonpharmaceutical interventions on OID transmission. Therefore, future research should explore the intersection between the COVID-19 pandemic and OID to gain a comprehensive understanding of the broader impact on public health.

### Conclusions

Our study revealed that the incidence of OID in Fujian Province exhibits a distinct distribution across populations, seasons, and regions. Notably, males and children aged >2 years are the most susceptible to OID. Moreover, our analysis demonstrated a shift in the seasonal peak of OID from autumn to winter or spring between 2005 and 2020. Our findings indicated that maximum temperature, atmospheric pressure, relative humidity, and precipitation positively influence the short-term incidence of OID. Zhangzhou City and Xiamen City were identified as the major hot spots for OID in Fujian Province. Therefore, it is essential to prioritize prevention and control efforts in these identified hot spots and among highly susceptible groups. Our findings provide valuable scientific evidence for policy makers to develop targeted intervention measures for OID, considering the specific disease pattern and geographical context.

## Appendix

Multimedia Appendix 1 Higher resolution version of Figure 3.

Multimedia Appendix 2 Higher resolution version of Figure 4.

## Abbreviations

AIC: Akaike information criterion
AICc: corrected Akaike information criterion
GAM: generalized additive model
GDP: gross domestic product
GLMM: generalized linear mixed model
GWR: geographic weighted regression
LISA: local indices of spatial association
OID: other infectious diarrhea
OLS: ordinary least regression

## Data Availability

The data sets analyzed during this study are available from the corresponding author upon reasonable request.

## References

[1] 2021 pneumonia and diarrhea progress report finds key gains despite toll of the COVID-19 pandemic. Johns Hopkins University.

[2] (2017). Diarrhoeal disease. World Health Organization.

[3] Liu H, Zhang J (2013). Analysis of the pathogens in infectious diarrhea (other than cholera, dysentery, typhoid and paratyphoid) cases reported in China in 2008. Zhonghua Yu Fang Yi Xue Za Zhi.

[4] Pathogen. Chinese Center for Disease Control and Prevention.

[5] Levy K, Woster AP, Goldstein RS, Carlton EJ (2016). Untangling the impacts of climate change on waterborne diseases: a systematic review of relationships between diarrheal diseases and temperature, rainfall, flooding, and drought. Environ Sci Technol.

[6] Chowdhury FR, Ibrahim QS, Bari MS, Alam MM, Dunachie SJ, Rodriguez-Morales AJ, Patwary MI (2018). The association between temperature, rainfall and humidity with common climate-sensitive infectious diseases in Bangladesh. PLoS One.

[7] Wang LP, Zhou SX, Wang X, Lu QB, Shi LS, Ren X, Zhang HY, Wang YF, Lin SH, Zhang CH, Geng MJ, Zhang XA, Li J, Zhao SW, Yi ZG, Chen X, Yang ZS, Meng L, Wang XH, Liu YL, Cui AL, Lai SJ, Liu MY, Zhu YL, Xu WB, Chen Y, Wu JG, Yuan ZH, Li MF, Huang LY, Li ZJ, Liu W, Fang LQ, Jing HQ, Hay SI, Gao GF, Yang WZ, Chinese Centers for Disease Control and Prevention (CDC) Etiology of Diarrhea Surveillance Study Team (2021). Etiological, epidemiological, and clinical features of acute diarrhea in China. Nat Commun.

[8] Mao Y, Zhang N, Zhu B, Liu J, He R (2019). A descriptive analysis of the Spatio-temporal distribution of intestinal infectious diseases in China. BMC Infect Dis.

[9] Phung D, Huang C, Rutherford S, Chu C, Wang X, Nguyen M, Nguyen NH, Do CM, Nguyen TH (2015). Temporal and spatial patterns of diarrhoea in the Mekong Delta area, Vietnam. Epidemiol Infect.

[10] Naumova EN, Jagai JS, Matyas B, DeMaria A, MacNeill IB, Griffiths JK (2007). Seasonality in six enterically transmitted diseases and ambient temperature. Epidemiol Infect.

[11] Kovats RS, Edwards SJ, Charron D, Cowden J, D'Souza RM, Ebi KL, Gauci C, Gerner-Smidt P, Hajat S, Hales S, Hernández Pezzi G, Kriz B, Kutsar K, McKeown P, Mellou K, Menne B, O'Brien S, van Pelt W, Schmid H (2005). Climate variability and campylobacter infection: an international study. Int J Biometeorol.

[12] Carlton EJ, Eisenberg JN, Goldstick J, Cevallos W, Trostle J, Levy K (2014). Heavy rainfall events and diarrhea incidence: the role of social and environmental factors. Am J Epidemiol.

[13] Pfeiffer DU, Robinson TP, Stevenson M, Stevens KB, Rogers DJ, Clements AC (2008). Spatial Analysis in Epidemiology.

[14] Sasson C, Cudnik M, Nassel A, Semple H, Magid D, Sayre M, Keseg D, Haukoos JS, Warden CR, Columbus Study Group (2012). Identifying high-risk geographic areas for cardiac arrest using three methods for cluster analysis. Acad Emerg Med.

[15] Jacquez GM, Greiling DA (2003). Local clustering in breast, lung and colorectal cancer in Long Island, New York. Int J Health Geogr.

[16] Laohasiriwong W, Puttanapong N, Luenam A (2017). A comparison of spatial heterogeneity with local cluster detection methods for chronic respiratory diseases in Thailand. F1000Res.

[17] Liu CP, Luo CL, Gao Y, Li FB, Lin LW, Wu CA, Li XD (2010). Arsenic contamination and potential health risk implications at an abandoned tungsten mine, southern China. Environ Pollut.

[18] Zhuang P, Zou B, Li NY, Li ZA (2009). Heavy metal contamination in soils and food crops around Dabaoshan mine in Guangdong, China: implication for human health. Environ Geochem Health.

[19] Alengebawy A, Abdelkhalek ST, Qureshi SR, Wang M (2021). Heavy metals and pesticides toxicity in agricultural soil and plants: ecological risks and human health implications. Toxics.

[20] Weng X, Wang Z, Ren J, Zhang Y, Yu L, Wang R (2019). Surveillance for public health emergencies caused by infectious diarrhea other than cholera, dysentery, typhoid and paratyphoid in China, 2014–2016. Dis Surveill.

[21] Fujian Province’s statutory reporting of infectious disease epidemics in September 2022. Fujian Provincial Health Commission.

[22] Hu WQ, Li YY, Ma W (2019). [Short-term impact of temperature on infectious diarrhea in southeast coastal area of China, 2005-2013]. Zhonghua Yu Fang Yi Xue Za Zhi.

[23] Chen KC, Lin CH, Qiao QX, Zen NM, Zhen GK, Chen GL, Xie YJ, Lin YJ, Zhuang SF (1991). The epidemiology of diarrhoeal diseases in southeastern China. J Diarrhoeal Dis Res.

[24] Diagnostic criteria for infectious diarrhea. National Health Commission of the People's Republic of China.

[25] Weather forecast page view statistics. RU: Reliable Prognosis.

[26] Wood SN (2017). Generalized Additive Models: An Introduction with R. 2nd edition.

[27] Thompson ES, Saveyn P, Declercq M, Meert J, Guida V, Eads CD, Robles ES, Britton MM (2018). Characterisation of heterogeneity and spatial autocorrelation in phase separating mixtures using Moran's I. J Colloid Interface Sci.

[28] Moran PA (1950). Notes on continuous stochastic phenomena. Biometrika.

[29] Chowdhury AI, Abdullah AY, Haider R, Alam A, Billah SM, Bari S, Rahman QS, Jochem WC, Dewan A, El Arifeen S (2019). Analyzing spatial and space-time clustering of facility-based deliveries in Bangladesh. Trop Med Health.

[30] Anselin L (1995). Local indicators of spatial association—LISA. Geogr Anal.

[31] Waller LA, Gotway CA (2004). Applied Spatial Statistics for Public Health Data.

[32] Kulldorff M, Athas WF, Feurer EJ, Miller BA, Key CR (1998). Evaluating cluster alarms: a space-time scan statistic and brain cancer in Los Alamos, New Mexico. Am J Public Health.

[33] Huang L, Kulldorff M, Gregorio D (2007). A spatial scan statistic for survival data. Biometrics.

[34] Rao H, Shi X, Zhang X (2017). Using the Kulldorff's scan statistical analysis to detect spatio-temporal clusters of tuberculosis in Qinghai Province, China, 2009-2016. BMC Infect Dis.

[35] Robertson C, Nelson TA, MacNab YC, Lawson AB (2010). Review of methods for space-time disease surveillance. Spat Spatiotemporal Epidemiol.

[36] Brunsdon C, Fotheringham AS, Charlton ME (2010). Geographically weighted regression: a method for exploring spatial nonstationarity. Geogr Anal.

[37] Akaike H (1974). A new look at the statistical model identification. IEEE Trans Automat Contr.

[38] Ordinary least Squares regression (OLS). XLSTAT: Your Data Analysis Solution.

[39] GILMOUR AR, ANDERSON RD, RAE AL (1985). The analysis of binomial data by a generalized linear mixed model. Biometrika.

[40] Vrieze SI (2012). Model selection and psychological theory: a discussion of the differences between the Akaike information criterion (AIC) and the Bayesian information criterion (BIC). Psychol Methods.

[41] (2019). RStudio: integrated development environment for R. KDnuggets.

[42] Yang X, Xiong W, Huang T, He J (2021). Meteorological and social conditions contribute to infectious diarrhea in China. Sci Rep.

[43] Chen C, Guan Z, Huang C, Jiang D, Liu X, Zhou Y, Yan D, Zhang X, Zhou Y, Ding C, Lan L, Lin Y, Wu J, Li L, Yang S (2021). Epidemiological trends and hotspots of Other Infectious Diarrhea (OID) in Mainland China: a population-based surveillance study from 2004 to 2017. Front Public Health.

[44] Wang H, Di B, Zhang T, Lu Y, Chen C, Wang D, Li T, Zhang Z, Yang Z (2019). Association of meteorological factors with infectious diarrhea incidence in Guangzhou, southern China: a time-series study (2006-2017). Sci Total Environ.

[45] Hongmei L (2020). Research on the epidemic characteristics and changing trends of other infectious diarrhea diseases in my country from 2005 to 2019. Chinese Center for Disease Control and Prevention.

[46] Luo HM, Ran L, Meng L, Lian YY, Wang LP (2020). [Analysis of epidemiological characteristics of report cases of rotavirus diarrhea in children under 5 years old in China, 2005-2018]. Zhonghua Yu Fang Yi Xue Za Zhi.

[47] Wu BS, Huang ZM, Weng YW, Chen FQ, Zhang YL, Lin WD, Yu TT (2019). Prevalence and genotypes of rotavirus a and human adenovirus among hospitalized children with acute gastroenteritis in Fujian, China, 2009-2017. Biomed Environ Sci.

[48] Zipfel CM, Colizza V, Bansal S (2021). The missing season: the impacts of the COVID-19 pandemic on influenza. Vaccine.

[49] Zhou X, Zhou Y, Chen R, Ma W, Deng H, Kan H (2013). High temperature as a risk factor for infectious diarrhea in Shanghai, China. J Epidemiol.

[50] Fang X, Liu W, Ai J, He M, Wu Y, Shi Y, Shen W, Bao C (2020). Forecasting incidence of infectious diarrhea using random forest in Jiangsu Province, China. BMC Infect Dis.

[51] Shope R (1991). Global climate change and infectious diseases. Environ Health Perspect.

[52] Chan MC, Mok HY, Lee TC, Nelson EA, Leung TF, Tam WW, Chan PK (2013). Rotavirus activity and meteorological variations in an Asian subtropical city, Hong Kong, 1995-2009. J Med Virol.

[53] Li W, Xiang W, Li C, Xu J, Zhou D, Shang S (2020). Molecular epidemiology of rotavirus A and adenovirus among children with acute diarrhea in Hangzhou, China. Gut Pathog.

[54] Chen C, Wu B, Zhang H, Li KF, Liu R, Wang HL, Yan JB (2020). Molecular evolution of GII.P17-GII.17 norovirus associated with sporadic acute gastroenteritis cases during 2013-2018 in Zhoushan Islands, China. Virus Genes.

[55] Guo YT, Hsiung CA, Wu FT, Chi H, Huang YC, Liu CC, Huang YC, Lin HC, Shih SM, Huang CY, Chang LY, Ho YH, Lu CY, Huang LM, Taiwan Pediatric Infectious Disease Alliance (2023). Clinical Manifestations and Risk factors of Campylobacter gastroenteritis in children in Taiwan. Biomed J (Forthcoming).

[56] Guo Z, Huang J, Shi G, Su C, Niu JJ (2014). A food-borne outbreak of gastroenteritis caused by norovirus GII in a university located in Xiamen City, China. Int J Infect Dis.

[57] Bao-jian C, Han-guo X, Rong-yan Z, Yan-rong L, Chen-xin L, Xian-liang X, Dian-wei J, Shan-ying Z (2018). Investigation and analysis of human intestinal protozoal diseases in Fujian province. Chin J Zoonoses.

[58] Environmental conditions of land-based sewage outlets into the sea and adjacent sea areas - 2009 Zhangzhou City. Marine Environmental Status Bulletin - China Marine Information Network.

[59] Thiagarajah JR, Kamin DS, Acra S, Goldsmith JD, Roland JT, Lencer WI, Muise AM, Goldenring JR, Avitzur Y, Martín MG, PediCODE Consortium (2018). Advances in evaluation of chronic diarrhea in infants. Gastroenterology.

[60] Hoshino Y, Kapikian AZ (1994). Rotavirus vaccine development for the prevention of severe diarrhea in infants and young children. Trends Microbiol.

[61] Black RE (1998). Therapeutic and preventive effects of zinc on serious childhood infectious diseases in developing countries. Am J Clin Nutr.

[62] Stauffer WM, Konop RJ, Kamat D (2001). Traveling with infants and young children. Part I: anticipatory guidance: travel preparation and preventive health advice. J Travel Med.

[63] Alebel A, Tesema C, Temesgen B, Gebrie A, Petrucka P, Kibret GD (2018). Prevalence and determinants of diarrhea among under-five children in Ethiopia: a systematic review and meta-analysis. PLoS One.

