# Supplementary figures and images for: Epidemic Characteristics, Spatiotemporal Pattern, and Risk Factors of Other Infectious Diarrhea in Fujian Province From 2005 to 2021: Retrospective Analysis
Source: JMIR Public Health Surveill. 2023 Nov 30;9:e45870. doi: 10.2196/45870 (PMC10722358; doi:10.2196/45870)

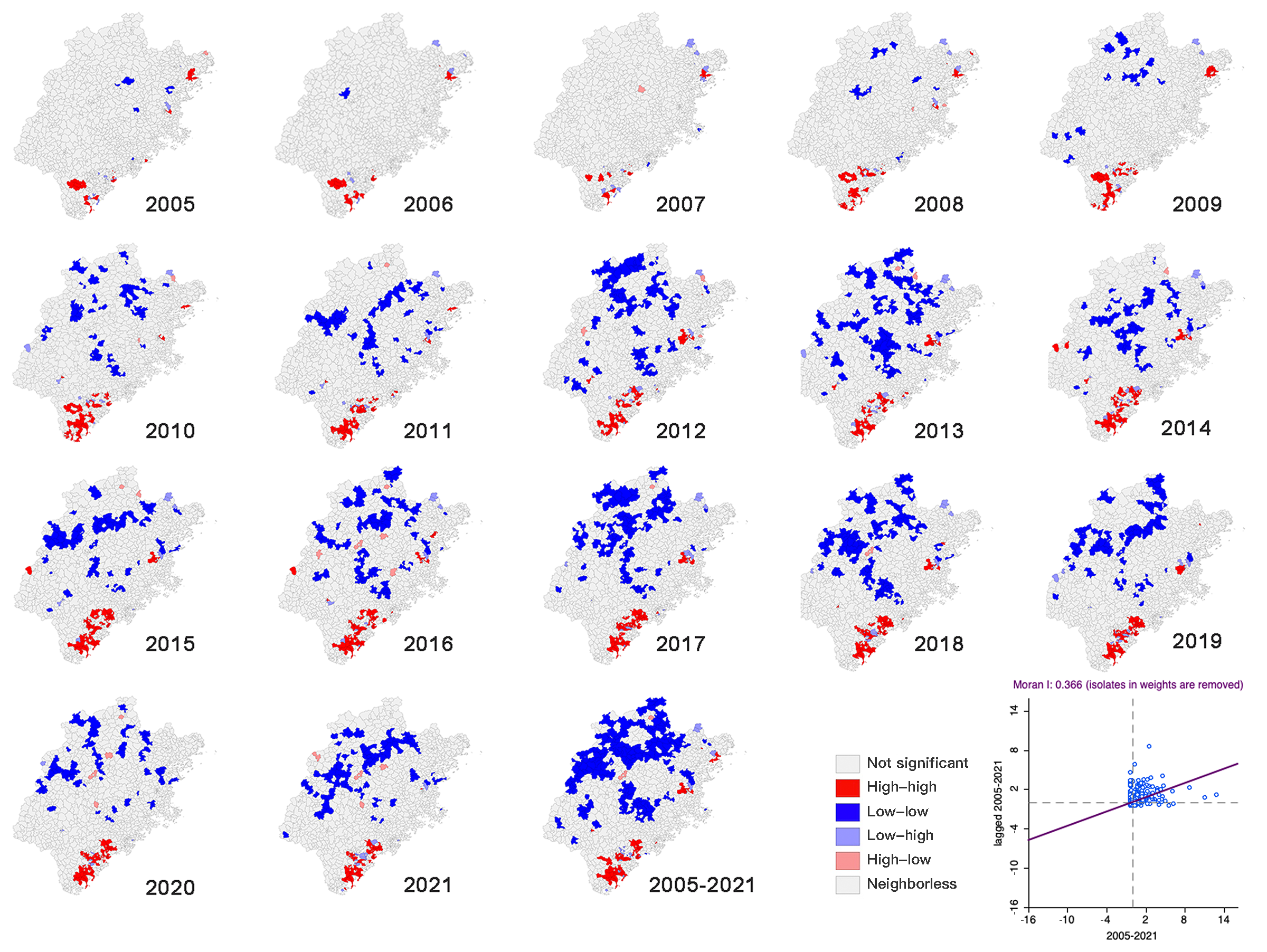

Supplement: Multimedia Appendix 1 [file publichealth_v9i1e45870_app1.png]

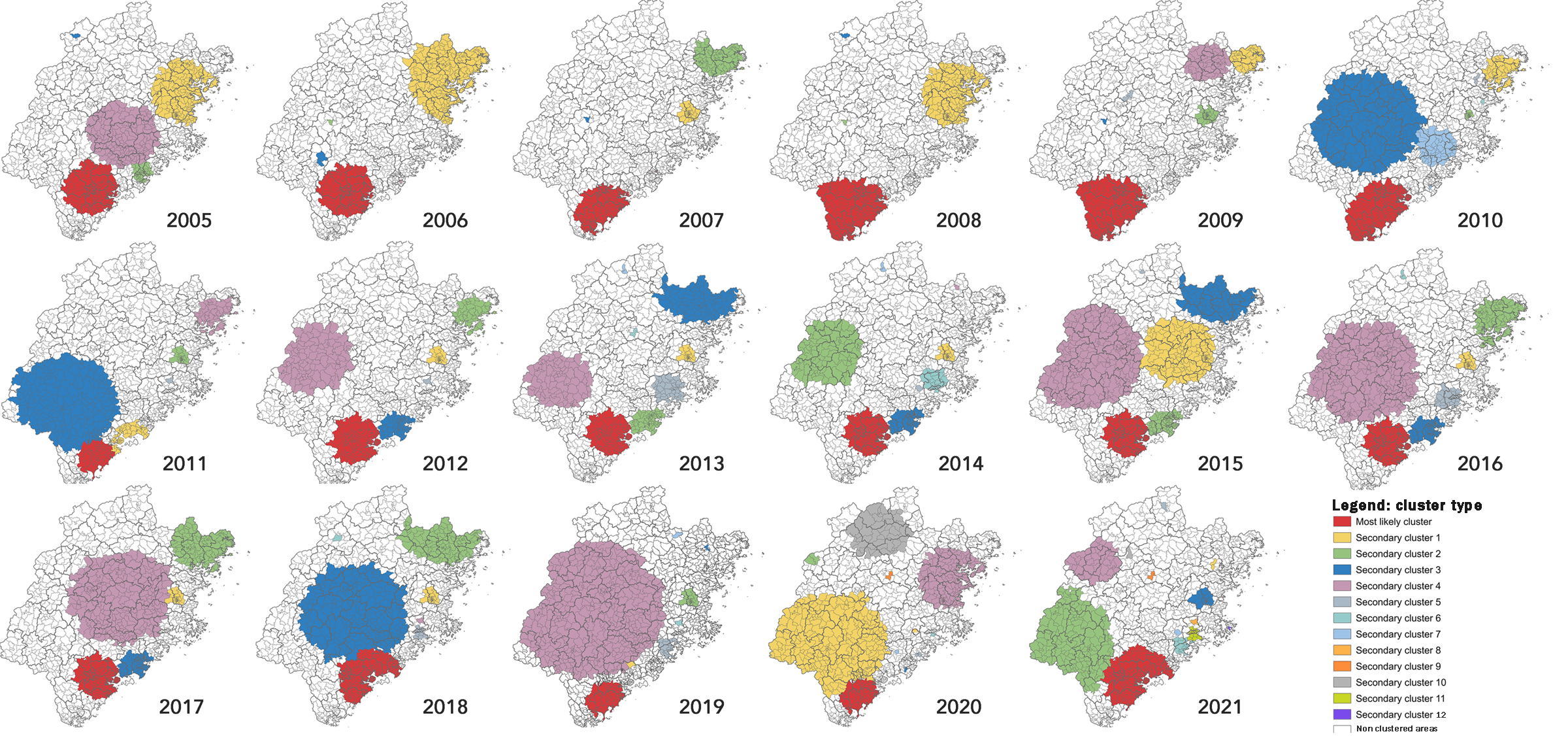

Supplement: Multimedia Appendix 2 [file publichealth_v9i1e45870_app2.png]
